# The Effect of Everolimus on Subependymal Giant Cell Astrocytoma (SEGA) in Children with Tuberous Sclerosis Complex

**DOI:** 10.22037/ijcn.v15i4.30591

**Published:** 2021

**Authors:** Hassan BAKHTIARY, mohammad BARZEGAR, Shadi SHIVA, Bita POORSHIRI, Parisa HAJALIOGHLI, Hamideh HERIZCHI GHADIM

**Affiliations:** 1 Pediatric Health Research Center, Tabriz University of Medical Science, Tabriz , Iran; 2 Department of Radiology , Tabriz University of Medical Science, Tabriz , Iran; 3 Department of Dermatology, Tabriz University of Medical Science, Tabriz , Iran

**Keywords:** Everolimus, Subependymal Giant Cell Astrocytoma, Tuberous Sclerosis Complex

## Abstract

**Objective:**

Subependymal Giant Cell Astrocytomas (SEGAs) are slow-growing glioneuronal tumors typically found around the ventricles of the brain, particularly near the foramen of Monro in 15%-20% of patients with tuberous sclerosis complex (TSC). Surgical resection is the standard treatment for these symptomatic tumors. The mTOR inhibitor everolimus can be regarded as an alternative treatment for SEGAs due to the complications of surgery. The present study primarily aimed to specify the effect of everolimus on SEGA volume change before and after treatment. The secondary objective was to determine the effect of this drug on renal angiomyolipoma (AML), skin lesions, and seizures in TSC patients.

**Materials & Methods:**

This pre- and post-treatment clinical trial was performed on 14 children (eight females and six males with a mean age of 10 years) previously diagnosed with TSC based on the diagnostic criteria. The subjects received oral everolimus at a dose of 3 mg/m^2^ for at least six months.

**Results:**

Half of the patients had more than 30% of volume loss in SEGA, and in 28.5% of them, a ≥ 50% reduction in SEGA volume was observed (P=0.01). Moreover, 92.9% of the patients had a ≥ 50% decrease in the frequency of seizures (P=0.000). The response rate in AML and skin lesions was 14.2% and 50%, respectively.

**Conclusion:**

Everolimus significantly reduced the seizure frequency and SEGA volume in the subjects; hence, it can be used as a potential alternative treatment for symptomatic SEGA in TSC patients.

## Introduction

Tuberous sclerosis, a genetic disorder with autosomal dominant inheritance, occurs in approximately 1/5800 live births (1). This disease is currently known as tuberous sclerosis complex (TSC) due to its probable effects on one or several other vital organs in addition to the central nervous system. This condition is characterized by the presence of hamartomas in many organs, especially the brain (such as cortical tubers, subependymal giant cell astrocytomas (SEGAs), and subependymal nodules), eyes, kidneys, skin, and heart (2, 3). 

Its clinical manifestations vary from mild cases with exclusive affection of the skin to life-threatening complications, such as hydrocephalous. The involvement of different organs is dependent on age; for instance, cardiac lesions usually occur in younger individuals, even in the fetal period, and renal lesions are often detected after adolescence. About one million people are affected by this disorder around the world (4). 

 Neurologic manifestations in TSC patients range from normal intelligence and the absence of seizures to intellectual disability and drug-resistant epilepsy. With a prevalence of 80-90%, the seizure is the most common neurologic sign in these patients (5-7). In these people, the seizure is mostly drug-resistant, and its control is significantly correlated with the cognitive performance of the patients (7, 8). There is no simple and easy test for TSC diagnosis. Although genetic studies have been proposed for this disease, diagnosis is currently based on clinical symptoms and paraclinical findings, and treatment depends on the signs (2). 

The major pathogenesis of tuberous sclerosis is the existence of tumor-like lesions (such as hamartomas) in diverse body organs, developed as a result of the mutation in either TSC 1 or TSC2 (9). Subependymal Giant Cell Astrocytomas (SEGAs) are slow-growing glioneuronal tumors typically found around the ventricles of the brain, particularly near the foramen of Monro in 15%-20% of the patients with TSC (3). The growth of this tumor is accompanied by a disturbed flow of cerebrospinal fluid, ensuing headache, vomiting, behavioral disorders, decreased sight, and sudden death from fatal acute hydrocephalous, and its risk is directly proportional to SEGA volume. These lesions do not regress spontaneously, but their volume increases progressively (2, 4, 10). 

Although surgery is the standard treatment for SEGA (5), complete resection is difficult due to the depth of these lesions along with the serious complications. Furthermore, tumor residues might lead to growth and relapse (10). Complete tumor resection was achieved in 66% of the patients, relapse was observed in 34% of the cases a year following the operation, and sequels of surgery occurred in 49% of the subjects (4). Consequently, it is only logical to evaluate effective non-surgical treatments. 

Recent studies (11, 12) have indicated the impact of the mammalian target of rapamycin (mTOR) inhibitor on SEGA regression in TSC patients. Based on the results of these studies, the Food and Drug Administration (FDA) has approved the application of this agent in TSC patients who are not suitable candidates for surgical resection (13). This approval has entailed the expansive usage of this medicine, and there is even consensus concerning the application of mTOR in patients with growing SEGA and no clinical signs. 

Everolimus is a rapamycin analog that reduces tumor volume through inhibiting mTOR in TSC patients (10). The present study primarily was done to specify the effect of mTOR on SEGA volume change before and after treatment. The secondary objective of this study was to determine the effect of this drug on renal angiomyolipoma (AML), skin lesions, and seizures in TSC patients. 

To our knowledge, there is no comprehensive data regarding the safety and efficacy of this drug in TSC children in Iran. 

## Materials & Methods

This prospective open-label, pre-, and post-treatment clinical trial was conducted on 17 patients previously diagnosed with TSC according to the diagnostic criteria in clinical and paraclinical evaluations. Via convenience sampling method, the participants were selected from those who were referred to the Children Neurology Clinic in the Pediatric Hospital of Tabriz affiliated with Tabriz University of Medical Sciences from August 2017 to March 2019. The inclusion criteria were at least one SEGA lesion larger than 0.5 cm in Magnetic Resonance Imaging (MRI) of the brain and willingness to enter the study.

The age range of the subjects was 3-19 years, with a mean age of 10 years. The paraclinical tests, including complete blood count (CBC), differential count, and biochemical tests for liver function tests, renal function, and lipid profile were performed at the beginning and six months after the treatment at the same center. Following initial assessments, everolimus (Afinitor, Novartis) was prescribed at a dose of 3 mg/m2. Drug titration and blood level measurement were not possible. The patients were visited by the same pediatric neurologist monthly and examined in terms of medication side effects and drug efficacy. 

Brain lesions were assessed by MRI at a power of 1.5 teslas at the beginning and six months post-treatment with the same apparatus and reported by the unit radiologist. The largest lesion diameter was measured using T1 VIBE fat-saturated images in axial, sagittal, and coronal slices. Afterward, the volume was calculated as follows: length × width × height/2. 

In some studies, a reduction of 30% or more and no new lesions during treatment were considered as the criteria for positive medication effects (14). However, in most studies, at least a 50% decrease was regarded for the positive effect of the drug on SEGA. In this study, we considered a reduction of ≥ 30% as a relative response and ≥ 50% as a favorable response. Seizure frequency was specified by a pediatric neurologist according to the clinical history and EEG at the beginning and six months after the treatment. During the observation period, AEDs remained unchanged, and everolimus was used as adjunctive, and baseline seizure frequency was assessed. A decrease of 50% or more in the frequency of seizures was considered favorable for this medicine. 

In renal AML, a reduction of 50% or more relative to AML volume in the baseline in the absence of new AML ≥1 cm and no AML-related bleeding of grade ≥ 2 was considered as a positive effect.

 This study was approved by the Ethics Committee of Tabriz University of Medical Sciences (IR.TBZMED.REC.1396.287 & IRCT20131012014988N2). Prior to treatment with everolimus, informed consent was obtained from both parents and patients (when possible). 

Data were analyzed using SPSS software version 21. The normality of data distribution was assessed by the Kolmogorov-Smirnov test. The qualitative, normal quantitative, and non-normal quantitative variables were described by frequency (percentage), mean (standard deviation), and median (25 and 75 percentile), respectively. To analyze the pre- and post-treatment data, McNemar's test was applied for the qualitative two-level variables, and the Sign test was utilized for the ordinal factors. A P < 0.05 was considered as the significance level for all tests. 

## Results

We evaluated 17 patients with SEGA in the present study. The treatment was ceased in one of the patients due to severe oral aphthous ulcers, and two patients left the study three months after its initiation. Ultimately, 14 patients (eight females and six males) were enrolled in this study for at least six months. Table 1 presents the baseline demographic data and the clinical findings of the TSC patients of different ages.

The mean age of the subjects was 10±3.3 years. All subjects had SEGA at baseline, and 71.4% had renal AML. Concerning other criteria, all patients had at least one skin lesion, 28.6% had retinal involvement, and cardiac involvement was observed in 35.5% of the patients.

**Fig1:**
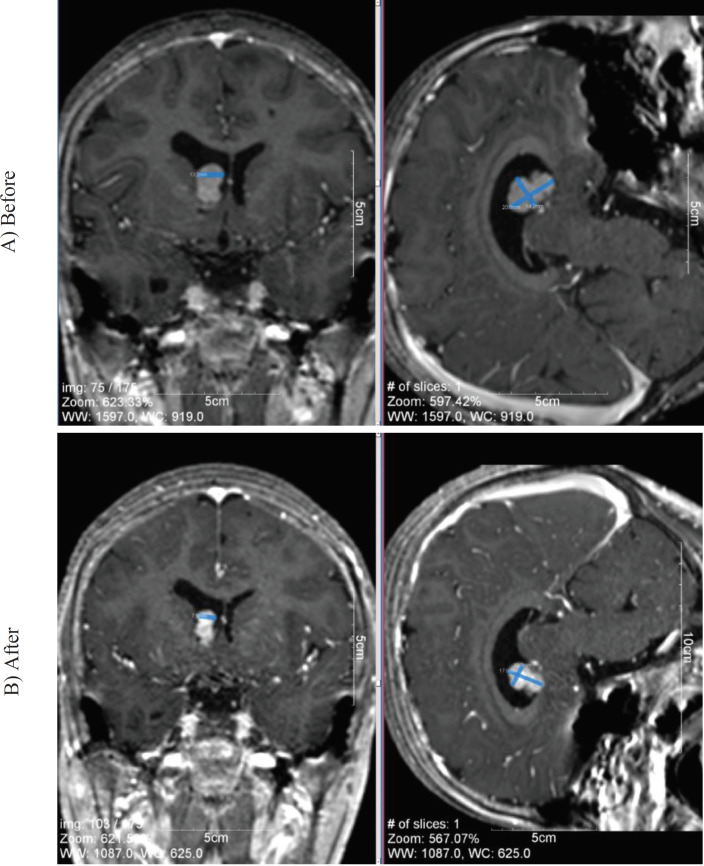
Changes in Subependymal Giant Cell Astrocytoma (SEGA) size before and after treatment by everolimus in patient number 5

In three patients (21.4 %), SEGA volume loss of 30% to less than 50% was noted; however, a reduction rate of 50% or more in SEGA volume was observed in 4 out of 14 (28.5%) patients (Fig.1). SEGA progression was detected in one patient (Case # 2, Table 1). The decrease in SEGA volume was statistically significant (P<0.01).

The medication was observed to be effective in seizure control. More specifically, the seizure was reduced by 90% and 50%-90% in 10 and three cases, respectively (P <0.001) (Table 2).

Out of 14 patients, 10 cases had AML at baseline, and only two patients (14.3%) responded to everolimus at 3 mg/m^2^.

All 14 patients had at least one skin lesion. Angiofibroma and hypomelanotic lesions were detected in 92.9% and 100% of the patients, respectively. Approximately half of the patients responded to the treatment. Facial angiofibromas decreased in size and became paler and less rough in 42.9% of the patients, but the number of lesions remained unchanged. The hypomelanotic macules regressed in size and color in half the patients, and no worsening of lesions was found during this study.

Although we did investigate the influence of this medication on the cognitive and behavioral performance of the patients, most parents were satisfied with the enhanced cognitive and behavioral performance of their children. 

*Medication Side Effects:* Symptoms of upper respiratory infection and pharyngitis were found in three patients; upon the emergence of these signs, the treatment was stopped and resumed after their resolution. However, one subject exhibited serious oral aphthous lesions and was excluded from the study. By stopping the drug, oral aphthous was resolved. 

## Discussion

The objective of this study was to evaluate the efficacy and safety of everolimus at a dose of 3 mg/m^2 ^in patients with TSC. Because we were not able to monitor the drug blood level, the treatment was commenced with a low drug dose. Meanwhile, after six months or more, the everolimus treatment resulted in a significant reduction in TSC symptoms and side effects.

Using the evaluated dose of everolimus, the SEGA response rate was 28.5%, and 21.4% of the patients showed noticeably reduced SEGA volumes. However, SEGA progression was detected in one patient who was a 13-year-old girl. SEGA progression rate was low (7.1%) in this study, which is in line with several studies, such as that of Trelinska et al. on 10 patients with TSC-related SEGA. They concluded that maintenance therapy with everolimus was an effective treatment method, which balanced the side effects for patients with TSC and SEGA following the standard treatment (10). 

Franz et al. (15) reported that everolimus significantly decreased the SEGA volume. Besides, Fogarasi et al. (16) confirmed the impact of everolimus in SEGA treatment. In another study, titled “Everolimus for SEGA: Final five-year Analysis”, Franz et al. (17) revealed that everolimus imposed a stable impact on SEGA tumor reduction after more than five years of treatment. 

In 2017, Arroyo et al. (18) conducted a study on a seven-year-old boy who had a large SEGA mass and immediately received everolimus. They reported that everolimus effectively reduced the tumor volume without the need for surgery. All the foregoing studies confirm the influence of everolimus on the treatment of SEGA caused by TSC. 

The present study mainly aimed at examining the effect of everolimus on SEGA, but we further evaluated the drug effect on other organs inflicted by TSC. A slight effect on renal angiomyolipoma and positive results associated with skin lesions were observed. The low response rate of renal angiomyolipoma might be attributed to the low drug dose and shortness of treatment. In most studies, treatment continued for more than 24 months. In the study carried out by Franz et al., the response rate was 54% after an average treatment duration of 28.9 months (19).

Based on the present study, in 42.9% of the patients, facial angiofibromas decreased in size and became paler and less rough. Also, the hypomelanotic macules regressed in size and color in 50% of the patients. Cie et al. demonstrated a skin lesion response rate of 37.5% after 12 months of therapy at a dose of 4.5 mg/m2 (20). Franz et al. observed a skin lesion response rate of 58.1% at 4.5 mg/m2 dose (21). Seemingly, skin lesion also responds to low doses of everolimus.

In TSC patients, the seizure is one of the most prevalent manifestations, usually occurring as infantile spasm during infancy (22). In this study, everolimus significantly lowered seizure frequency in TSC patients. Many studies are consistent with the present research, confirming the efficacy of everolimus in reducing seizure frequency in TSC patients (8, 23-31).

Mizuguchi et al. (32) and Hwang et al. (33) observed notable improvement in behavior and autistic symptoms in patients but we did not control it by questionnaire.

**Table1:** Baseline demographic and clinical findings of the enrolled patients

Pt.nr./ gender	Age at drug start	Epilepsy	SEGA	SEN	Hypopigmented macules	Angiofibroma	AML	Cardiac lesions
1/M	19	Yes	Yes	Yes	Yes	Yes	Yes	No
2/F	13	Yes	Yes	No	Yes	Yes	Yes	No
3/F	8	Yes	Yes	Yes	Yes	Yes	Yes	Yes
4/M	10	Yes	Yes	Yes	Yes	No	Yes	No
5/F	14	Yes	Yes	Yes	Yes	Yes	Yes	No
6/F	8	Yes	Yes	Yes	Yes	Yes	No	No
7/F	10	Yes	Yes	Yes	Yes	Yes	No	No
8/M	7	Yes	Yes	Yes	Yes	Yes	Yes	Yes
9/M	10	Yes	Yes	Yes	Yes	Yes	Yes	No
10/M	9	Yes	Yes	Yes	Yes	Yes	No	No
11/F	10	Yes	Yes	Yes	Yes	Yes	Yes	Yes
12/F	6	Yes	Yes	Yes	Yes	Yes	Yes	Yes
13/M	9	Yes	Yes	Yes	Yes	Yes	No	No
14/F	7	Yes	Yes	Yes	Yes	Yes	Yes	Yes

AML, Angiomyolipoma ; F, Female ; M, Male ; mo,months ; nr., Number ; Pt., Patient ; SEGA, Subependymal Giant Cell Astrocytoma ; SEN, Sub Ependymal Nodules.

**Table2:** Effect of Everolimus on Epilepsy

**Treatment response** ** (decrease seizure frequency %)**	**Seizure frequency/ month after everolimus**	**Seizure frequency/ month ** ** before everolimus**	**Seizure type( Habitual) **	**Number of AEDs trial**	**Age of seizure onset**	**Age** **(year)/** **Gender**	**N#**
90˃	Seizure Free	4-5	Focal to bilateral tonic clonic	5 (PB, VGB, CLB, VPA, CBZ)	3y	19 /M	**1**
90˃	Seizure Free	6	Focal without awareness motor/ tonic	5 (VPA, VGB, CBZ, CLB, ACZ)	3y	/F 13	**2**
90˃	Seizure Free	10	Focal without awareness/non-motor	5 (VPA, VGA, PB, LTG, CLB)	2y	/F 8	**3**
50˃	12	30	Drop attack	6 (VPA, PB, VGB, CLB, LTG, ETX)	7m	/ M 10	**4**
90˃	1	11-12	Generalized Tonic	5 (VPA, VGA, CBZ, PHT, CLB)	4y	/F 14	**5**
50˂	45	60	Epileptic Spasm	5 (PB, VGB, CLB, ACTH, NZP )	1m	/F 8	**6**
90˃	2	25	Generalized myoclonic	6 (VPA, VGA, PRM, CLB, LTG, ACTH)	4m	/F 10	**7**
90˃	Seizure Free	8	Focal without awareness /motor Clonic	5(VPA, PB, CLB, CBZ, LTG)	2m	/ M 7	**8**
90˃	1	15	Focal without awareness/ motor tonic	5 (PB, CLB, VGB, VPA, LTG)	1.5y	/M 10	**9**
90˃	Seizure Free	6	Focal without awareness/ Tonic	4 (CLB, CBZ, VGB, PB)	2y	/ M 9	**10**
50˃	40	100	Drop attack	5 (CBZ, VGB, LTG, PB, CLB)	9m	/F 10	**11**
50˃	20	60	Tonic Spasm	5 (CBZ, VGB, CLB, ACTH, PB)	4m	/F 6	**12**
90˃	4	45	Focal to bilateral tonic clonic	5 (CBZ, VGA, PRM, PB, LTG)	5m	/ M 9	**13**
90˃	5	55	focal without awareness/ non motor /emotional /gelastic	5 (CBZ, PB, CLB,VGB,VPA)	2m	/F 7	**14**

ACTH: Adrenocorticothropin hormone; ACZ: Acetazolamide; AED: Anti-Epileptic Drugs; CBZ: Carbamazepin; CLB: Clobazam; ETX: Ethosuximide; F: Female; GTC: Generalized Tonic Clonic; LTG: Lamotrigine; M: Male; NZP: Nitrazepam; PB: Phenobarbital; PRM: Primidone; VGB: Vigabatrin; VPA: Valproic Acid

## Limitations of this study: 

No genetic studies for mutations of TSC1 and TSC2, small sample size, and short follow-up due to high cost of the drug, no checking for drug level due to unavailability to do in our center. 

## In Conclusion

According to the findings of this study, everolimus significantly affected the multisystem manifestation of TSC. More specifically, this drug reduced the seizure frequency and SEGA volume in TSC patients, which can be a safe alternative treatment for TSC patients with SEGA. 
